# The coinfection of ALVs causes severe pathogenicity in Three-Yellow chickens

**DOI:** 10.1186/s12917-024-03896-1

**Published:** 2024-02-01

**Authors:** Peikun Wang, Jing Wang, Na Wang, Cong Xue, Zhaoqing Han

**Affiliations:** 1 Institute of Microbe and Host Health, Linyi University, Linyi, 276000 Shandong China; 2 Animal Epidemic Disease Anticipatory Control Center, Lanshan District, Linyi, 276005 Shandong China

**Keywords:** Coinfection, Monoinfection, Avian leukosis virus, Pathogenicity, Three-yellow chickens

## Abstract

The coinfection of ALVs (ALV-J plus ALV-A or/and ALV-B) has played an important role in the incidence of tumors recently found in China in local breeds of yellow chickens. The study aims to obtain a better knowledge of the function and relevance of ALV coinfection in the clinical disease of avian leukosis, as well as its unique effect on the pathogenicity in Three-yellow chickens. One-day-old Three-yellow chicks (one day old) were infected with ALV-A, ALV-B, and ALV-J mono-infections, as well as ALV-A + J, ALV-B + J, and ALV-A + B + J coinfections, via intraperitoneal injection, and the chicks were then grown in isolators until they were 15 weeks old. The parameters, including the suppression of body weight gain, immune organ weight, viremia, histopathological changes and tumor incidence, were observed and compared with those of the uninfected control birds. The results demonstrated that coinfection with ALVs could induce more serious suppression of body weight gain (*P* < 0.05), damage to immune organs (*P* < 0.05) and higher tumor incidences than monoinfection, with triple infection producing the highest pathogenicity. The emergence of visible tumors and viremia occurred faster in the coinfected birds than in the monoinfected birds. These findings demonstrated that ALV coinfection resulted in considerably severe pathogenic and immunosuppressive consequences.

## Introduction

Globally, avian leukosis viruses (ALVs) cause severe economic losses to the poultry industry because of their infectiousness and retroviral properties [1]. Based on the host range [2], antibody neutralization and receptor interference studies [3], ALVs that infect chickens can be divided into subgroups A, B, C, D, E, J and K. Among these subgroups, subgroup J (ALV-J), which was first isolated from meat-type breeder chickens in 1988 [4], is the most prevalent in chickens [5–7]. Following the initiation of a nationwide eradication program (NEP) in 2008, the infection rate of exogenous ALVs in China decreased significantly [8]. However, the infection and clinical problems of ALV-A, ALV-B and ALV-J are still common in local chickens [9].

Besides the economic impact of ALV mono-infections [10, 11], epidemiological studies have demonstrated that ALVs commonly co-infect each other [12]. In our previous study, approximately 44.44 percent of commercial yellow chickens in southern China were infected with ALV monoinfection or coinfection [9]. Furthermore, it also showed that MDV and ALV coinfection caused greater economic losses than MDV monoinfection in Chickens [8]. Chickens infected with REV and ALV-J showed more severe growth retardation and immunosuppression [13].

Simultaneous infections with more than one subgroup in the same chicken have rarely been previously reported for ALV. Our previous studies demonstrated that simultaneous infections with ALV-A, ALV-B, and ALV-J (ALV coinfection) occurred in a Three-yellow chicken that experienced severe tumors in the clinic [9]. The purpose of this investigation was to understand more about the function and relevance of ALV coinfection in clinical avian leukosis, as well as its influence on pathogenicity in three-yellow chickens.

## Materials and methods

### Virus

The ALV-A strain GX14DJ3-18(Accession No. MH213216) [14], ALV-B strain GX14FF03(Accession No. KU923579) and ALV-J strain GX15MM6-2 (Accession No. KU934276) were all isolated from local breed native chickens and kept in our laboratory [15].

### Birds and animal experiment

A total of 280 1-day-old ALV-negative Three-yellow chicks (purchased from an AL-negative breeding flock, the chicks were tested again upon arrival with the meconium to ensure sure they were indeed free of ALV infection by ELISA) were randomly divided into seven groups (*n* = 40/each). All chickens were inoculated intra-abdominally at 1 day of age with ALV-A 10^4^ TCID_50_ per bird alone (Group A), ALV-B 10^4^ TCID_50_ per bird alone (Group B), ALV-J 10^4^ TCID_50_ per bird alone (Group J), both ALV-A and ALV-J 10^4^ TCID_50_ per bird (group AJ), both ALV-B and ALV-J 10^4^ TCID_50_ per bird (group BJ), and both ALV-A, ALV-B and ALV-J 10^4^ TCID_50_ per bird (group ABJ). To mitigate the potential impact of varying viral quantities on experimental outcomes, a standardized viral inoculation of 10^4^ TCID_50_ for the total virus included in coinfection group. The chickens from the control group were inoculated with DMEM (40 birds, control group). Birds of different groups were isolated and provided with formulated feed and drinking water ad libitum. In all experiments involving animals, protocols were evaluated and approved by Linyi University's Animal Experimental Ethical Inspection Form. Chickens were terminally anaesthetised by CO_2_, and decapitated.

### Measuring the weights of the body, immune organs and viremia of the experimental birds

To study the effect of ALVs monoinfection and/or coinfection on body and immune organ weights, 5 birds were randomly selected from each group to be weighed at 1, 3, 5, 8, 11, and 15 wpi. Following necropsy, the major avian immune organ (bursa offabricius,spleen, thymus) were sampled and weighed to determine the immune organs' relative weights. At 1, 3, 5, 8, 11 and 15 wpi, viremia was determined in 10 birds from each group, blood samples were obtained for virus isolation, and plasma was separated as described previously [16]. Briefly, the plasma was inoculated into the DF-1 cells grown in a 96-well plate and then the cultures were grown for 7 d before they were used for ALV-P27 antigen detection with an ELISA kit (Biochek, Holland).

### Gross and histological lesions

A count was carried out on the number of birds with tumor lesions in the groups of infected birds. We collected organs with gross tumors (like liver, kidney, spleen). Then the tissues were fixed in neutral buffered formalin, embedded in paraffin, sectioned, routinely stained with hematoxylin and eosin [17]. The pathological sections were observed using an optical microscope (ECLIPSE 80i Nikon, Japan).

### Statistical analysis

For statistical analysis, SPSS Windows Version 22 software (consisting of at least three repeated data groups) was used. The data were also processed with GraphPad Prism (8.4.2) and expressed as mean ± SE. The differences between the groups were assessed using one-way ANOVA analysis. The differences among groups were considered extremely significant (*P* < 0.01) and otherwise significant (*P* < 0.05).

### Results and discussion

**Fig. 1:**
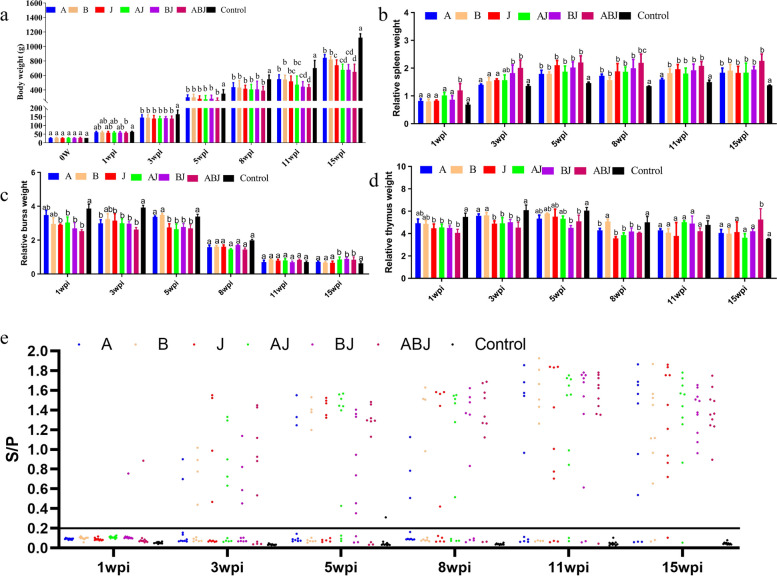
Influences of ALV infection on body weight (**a**), spleen relative weight (**b**), bursa relative weight (**c**) and thymus relative weight (**d**) in Three-Yellow chickens at 1, 3, 5, 8, 11 and 15 wpi. Five birds were randomly picked from each group to be weighed. The mean weight, which are followed by different lower-case letters, was significantly different (*P* < 0.05) based on Duncan’s multiple range test (X ± SE). **e** ALV-positive identification by ALV P27 antigen ELISA; an S/P value greater than or equal to 0.2 was regarded as positive

As demonstrated in Fig. 1a, the mean weights of the birds in Group ABJ were clearly lower than those in the control group at 1 wpi. (*P* < 0.05). The mean weights of the birds in all treatment groups were significantly (*P* < 0.05) lower than the mean weights of the birds in the control group at 3 wpi, as previous studies have shown [14]. However, at 15 wpi, the mean weights of the birds in the ALV-ABJ coinfection group were considerably (*P* < 0.05) lower than those in the ALV monoinfection group. As previously stated, ALV-A-, ALV-B-, and ALV-J-infected groups all showed progressive emaciation, and ALV coinfection might produce a more severe reduction in body weight gain.

Following necropsy, the bursa offabricius, spleen, thymus were taken and weighed to estimate the indices of relative weight of immune organs (RWIO) and immune organ weight (g) of bird body weight (kg). Overall, the spleen index of the infection group was higher than that of the control group (Fig. 1b), while the bursa of Fabricius and thymus index of the infection group were lower than those of the control group (Fig. 1c, d). The spleen of the challenge group was enlarged throughout the experiment, whereas the bursa of Fabricius and thymus were mainly concentrated in the early stage of the experiment. These trends of RWIO for the spleen, thymus, and bursa were similar to the trends reported in previous studie [8]. The spleen index of the coinfection group was greater than that of the ALV monoinfection group, according to further study. In the early phases of the experiment, atrophy of the thymus and bursa was greater in the coinfection group than in the monoinfection group. These findings suggest that ALV coinfection is more pathogenic than ALV monoinfection.

At 1 wpi, one bird in each group BJ and ABJ developed viremia, as shown in Fig. 1e. All challenged groups had viremia at 3 wpi, and from 5 to 15 wpi, the coinfection group had a greater viremia-positive rate than the monoinfection group. These findings suggested that ALV coinfection resulted in earlier and more severe viremia than ALV monoinfection.

**Fig. 2:**
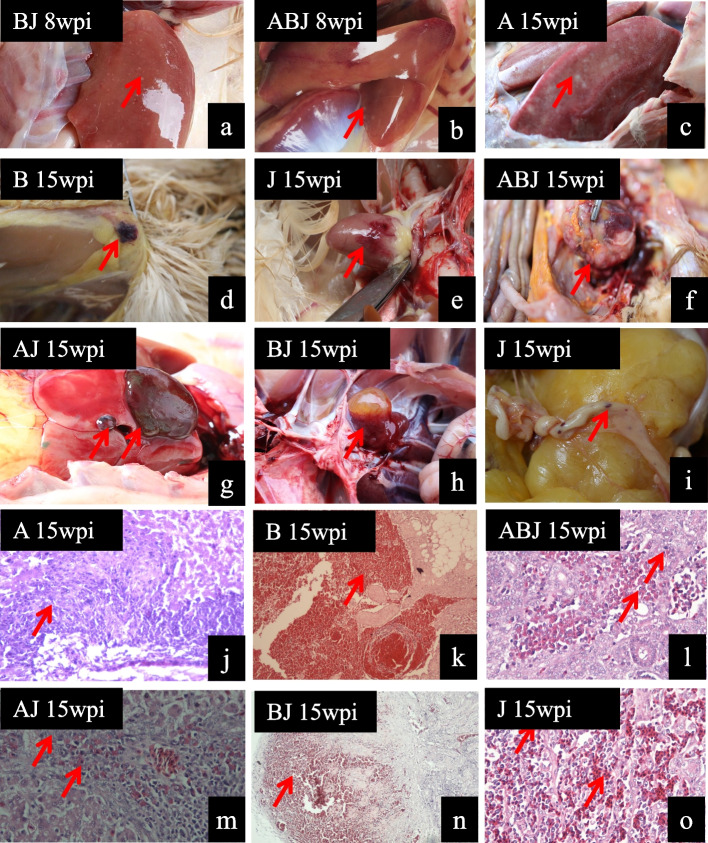
Anatomical and histopathological lesion results. **a** white tumor nodules on the liver surface; **b** mottled hemorrhage on the liver surface; **c** white tumor nodules on the liver surface; **d** hemangioma in subcutaneous locations; **e** hemangioma on the heart; **f** tumor in the abdomen; **g** hemangioma on the liver; **h** hemangioma on the kidney; **i** hemangioma on the intestine; **j** numerous proliferating tumor cells were concentrated in the hepatic tissue; **k** blood cells in the subcutaneous locations hemangioma region; **l** numerous infiltrations of myelocytes were found and that were characterized by acidophilic granules in the cytoplasm, this is a further microscopic observation for tumor in Fig. 2f; **m** Myeloblasts and Lymphocytes formed proliferation focus in liver; **n** blood cells in the kidney hemangioma region; **0** blood cells in intestine hemangioma region

There were no deaths in any of the groups during the whole experiment. At 8 wpi, the first case of clinical tumor was seen in the ALV coinfection group, presenting as white tumor nodules with speckled bleeding on the liver surface (Fig. 2a, b). Following autopsy, all infected birds in the ALV coinfection group and the ALV monoinfection group had gross histopathological lesions, with some having white tumor nodules on the liver surface (Fig. 2c), hemangioma on the subcutaneous locations (Fig. 2d), hemangioma on the heart (Fig. 2e), hemangioma on the liver (Fig. 2g), hemangioma on the kidney (Fig. 2h),and hemangioma on the intestine (Fig. 2i). In the ALV-A/B/J coinfection group, an abdominal tumor was discovered (Fig. 2f). The hepatocytes of ALV-A infected birds were squeezed by numerous proliferating tumor cells (Fig. 2j). As expected, there were many blood cells and heterophilic lymphoid cells in the hemangioma (Fig. 2k, l, m, n, o).

ALV-A and ALV-B predominantly induce lymphoid leukosis, encompassing hemangioma and diverse cell tumor types. Conversely, ALV-J primarily elicits myeloid leukosis, with a higher prevalence of vascular neoplasms [12]. Our study further revealed the occurrence of lymphoid leukosis resulting from ALV-A infections (Fig. 2c, j), vascular neoplasms arising from ALV-B infections (Fig. 2d, k), and hemangioma caused by ALV-J infections (Fig. 2e, o). Within the coinfection group, hemangioma were predominantly observed. Additionally, the ABJ coinfection group exhibited the presence of large tumors in the abdominal cavity. According to these findings, we hypothesized that coinfection primarily arises from tumor types induced by ALV-J, while A and B subgroup viruses contribute to augmenting the pathogenicity of J subgroup viruses. This observation aligns with the established notion that J subgroup viruses exhibit a greater degree of pathogenicity compared to A and B subgroup viruses [15].

**Fig. 3:**
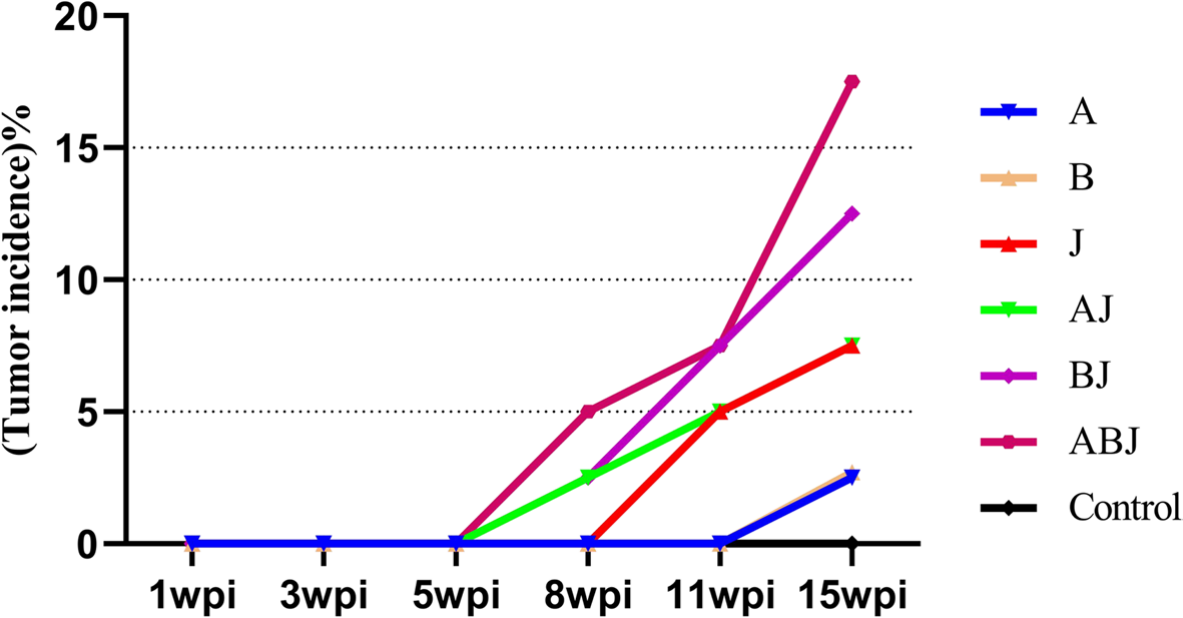
Comparison of ALV infections on tumor incidence in Three-Yellow birds challenged

Following the challenge, the chicken tumorigenesis in each challenge group was observed, along with the pathology section results, and the chicken tumorigenesis was tallied and documented. At 15 wpi, the tumor incidence order was group ABJ (17.5%) > group BJ (12.5%) > group AJ (7.5%) = Group J (7.5%) > Group B (2.5%) = Group A (2.5%), as illustrated in Fig. 3. These findings suggested that ALV coinfection was more tumorigenic than ALV monoinfection.

Prior studies have shown that the number of coinfection tumorigenic virus cases has grown, causing significant damage to the poultry industry. Coinfection with REV and ALV-J, for example, causes more serious mortality, growth retardation, and immunosuppression in SPF chickens [13]; coinfection with MDV and REV reduces MD vaccine [18]; and coinfection with MDV and ALV-J caused greater economic losses, growth retardation, and immune organ damage [8]. This study showed that ALV coinfection induced more pathogenic effects, growth arrest, and immune organ damage in Three-Yellow Chickens than ALV monoinfection.

Remarkably, despite the potential for causes severe pathogenicity due to ALV coinfection, no fatalities were recorded during the entire course of the experiment. This outcome aligns with expectations, as our previous research has demonstrated that infection with these three viruses individually does not result in chicken mortality, indicating their low lethality [14, 15]. Furthermore, the age of the challenged chicken is a pertinent factor. In this study, the chickens were euthanized at 15 weeks of age. In the clinical case, the mortality age of three-yellow-chickens afflicted with ALV typically at a later stage. Just as the three strains used in this study were isolated from 140, 133, and 120 days sick chickens, respectively [14, 15]. Consequently, it is plausible to hypothesize that prolonging the rearing period of these infected chickens could potentially result in their demise.

The presence of coinfection of distinct ALV subgroups in the field may give a possibility for viral gene recombination among the different ALV subgroups, in addition to boosting the pathogenicity of chickens. The original ALV-J isolate, for example, has been linked to recombination between an exogenous virus and an endogenous retroviral sequence [19], as have JS15SG01 [20], DL00766 [21], BR119 [22], HB2015032 [23], and others. Viral recombination might be the product of spontaneous virus evolution aimed at virus dissemination [24]. The foundation of viral recombination was the simultaneous infection of ALVs in the same cells.

Briefly, there are no effective drugs or vaccinations available to treat or suppress ALV outbreaks. The general management strategy for this infection is to eradicate all exogenous ALV and should be the responsibility of the primary breeder. Otherwise, detecting and treating coinfections becomes increasingly challenging.

## Data Availability

The data presented in this study would be available on request from the corresponding author. All data related to this study is part of a thematic research which is ongoing and due to this fact data are not publically available.
